# Approaching collaboration in primary care differently: Exploring boundaries and boundary objects

**DOI:** 10.1177/08404704241271150

**Published:** 2024-08-13

**Authors:** Monique Walsh

**Affiliations:** 1 97950 University of British Columbia, Kelowna, British Columbia, Canada.

## Abstract

In recent primary care policy, collaboration is often understood as an outcome, such as the delivery of team-based care or an integrated health system. This outcome-based understanding of collaboration in policy has proven challenging to achieve in practice. This article introduces the concepts of constructing boundaries and boundary objects used in other disciplines, to support our understanding of collaboration by observing the collaborative process. Multiple methods, such as semi-structured interviews, discourse analysis, and member-checking, were used to compare primary care collaborations across three distinct time periods during the onset of COVID-19 within Interior British Columbia. Data analysis revealed the changing nature of boundaries and boundary objects, providing insights into the collaborative process. Through the exploration of boundaries and boundary objects, this article provides a way to approach collaboration in practice differently. By better understanding the process of collaboration, this research could potentially improve collaborative outcomes.

## Introduction

Across primary care workplaces, healthcare professionals, as well as the administrative and executive teams that lead and support them, are continually being called upon to collaborate. Collaboration can be understood as both the *process* and the *outcome* of coming together, building, crossing, and re-creating new norms and expectations. However, in healthcare policy documents, collaboration is often defined as the outcome of clinical delivery of care, such as team-based care or of an integrated health system.^1-4^ This outcome-based understanding of collaboration in policy has proven challenging to achieve in practice. In this article, I focus on the *process* of collaboration, specifically in primary care workplaces in which administrative, executive, and clinical team members collaborate between themselves and their environment in both clinical and non-clinical contexts. In doing so, I will share two concepts from other fields that can foster a process view of collaboration: (1) the construction of boundaries and (2) the construction of boundary objects.

“Boundaries” exist all around us and they simultaneously enable and restrict different types of interactions. Boundaries are negotiated through practice, whether intentionally or unintentionally. This ongoing negotiation of the boundary, “the location of a relationship where the relationship both separates and connects,”^5^ means that a boundary includes some possibilities and excludes others. The different constructed boundaries explored in this research include, for example, changes to clinical space due to COVID-19, physical distancing requirements, scopes of practice between providers, and uses of technology.

“Boundary objects” are objects used to bridge boundaries as a result of the need to communicate, but their use does not necessarily require shared understandings or desires. They are continually changing and are created through practice. As boundaries evolve and change, the objects used for knowledge-sharing across these boundaries also change.

Tracing boundaries and boundary objects in the workplace allow researchers to better understand organizational structures, work as practice, and how people learn and know within their workplaces. One example of mapping boundaries occurred within an on-line academic setting; this research made visible the informal learning that was happening in that workplace. With this information, the educational organization was able to adapt their support structures accordingly.^6^ An example of using boundary objects as a way to better understand process is a study that traced how a contract moved through an organization for signature.^7^ This study made the knowledge practices within that organization visible and therefore changeable.

### Collaborative primary care policy

Over the last 10 years, provincial policy regarding primary care in British Columbia (BC) as in other jurisdictions, has emphasized a key theme of collaboration. This includes inter-collaborations by interdisciplinary providers within a single team, intra-collaborations across teams of care providers, as well as broad organizational collaborations, such as with health authorities and community partners. *The Primary and Community Care in BC: A Strategic Policy Framework*^8^ identified Patient Medical Homes and linkages across sub-specialities, specialities, community, and allied health as critical. Since 2015, BC’s Ministry of Health has issued a series of policy discussion papers on community-based programs including primary care networks, urgent care centres, specialized community services programs, and community health centres. One aspect these papers have in common is that they all emphasize collaborative expectations.

Recognizing the large-scale challenges of requiring collaboration across providers, disciplines, and partners could be one of the reasons that we see more emphasis placed on interdisciplinary and interprofessional work.^9^ This focus helps to develop tangible practices that deliver on collaborative policy direction and aligns with the quintuple aim” of patient satisfaction, provider satisfaction, cost efficiency, the health of the population, and health equity.^10-12^ However, as Brandt^13^ reminds us, this focus on high-performing collaborative care teams means that the field of health must look to other sectors, including management and education, for new approaches.

In order to address a practical need for healthcare professionals to implement collaborative healthcare transformation, I asked: How does the awareness of the *process* view of collaboration prompt new ways of approaching collaborations in primary care workplaces?

## Methods

To inform this research, I used multiple methods for data collection to document collaborations across three dynamic time periods: (1) the early, (2) the middle, and (3) the later stages of the first wave of COVID-19 experienced in BC. The first phase of data collection included virtual semi-structured interviews with 15 participants to identify collaborative processes experienced during three separate time periods: January-March 2020, March-May 2020, and June-August 2020 across primary healthcare delivery. These participants included a mix of administrative, clinical, and executive personal experiences of working in primary care. Phase 2 of data collection included the analysis of publicly sourced documents in order to trace the collaborative processes over time. This included a review and analysis of provincial orders, modelling, and health acts. The last phase of data collection involved checking the data with the participants, the so-called member-checking. The purpose of member-checking was three-fold. First, I reviewed the observed changes to the collaborative processes over time with participants. Second, I reviewed with participants the coding and theming from the various data sources used. Third, participants were given the chance to review their own quotes and provide any further clarifications if needed. Participants shared questions they had, additional considerations for this work, and discussion on how they might apply the work.^14-16^

## Constructing boundaries and boundary objects

### Boundaries

So why boundaries? To begin observing boundaries in our collaborative processes we first have to understand what they are. Boundaries are really the point of a relationship that simultaneously bring the parties together and create a distance.^5^ Boundaries can be seen at the individual level, professional level, or organizational level (both internal and external, horizontal and vertical); they are happening simultaneously and are often multifaceted. The concept of a boundary may invoke imagery of concreteness, but, in fact, the edges on which boundaries exist are being constantly created and adapted.^5^ As Marshall describes, “people are constantly parcelling up their worlds, and having their worlds parcelled up for them, by a series of distinctions between inside and outside, identity, and difference.”^17^

Sometimes boundaries in healthcare workplaces can be a source of tension, such as silos between departments in a hospital setting or varying understanding regarding the scopes of practice within an interdisciplinary care team. However, sometimes boundaries can be enabling. For example, they can be experienced as an increase of personal autonomy and support empowerment of team members through clear roles and responsbilities.^18^ Boundaries can enable action to happen at times or limit it.^5,6^ The place of opportunity for communication and bridging is really how we can begin to see the construction of boundaries and collaboration as synonymous.^19,20^

Although there are alternatives, I categorized boundaries in my research using Hernes’ three types of boundaries: (1) physical, (2) social, and (3) mental.^6^

Boundaries are not pre-determined or stable; they are created through the coming together of different people and objects across time. They are created in practice. Working across boundaries and consistently redefining them is the collaborative work that must happen in workplaces, especially in primary care (Tables 1-3).

**Table 1.** Hernes’ categories of boundaries^1,6^.

Category	Description	Example	Purpose
Physical	Includes both material and regulatory boundaries	Walls or roads	Impose real constraints while at the same time enabling
		Rules, guidelines, scope of practice	
Social	Includes ways of distinguishing identity	Formal, professional boundary between physician and patient	Create inclusion, expertise, trust, and solidarity while simultaneously creating exclusion and alienation
		Informal boundary, such as social networks and professional contacts	
Mental	Includes subjective separations in our own minds as to what goes together and what does not	A stereotype about a certain patient or groups of patients	Can help to make sense of the world
			Focuses on certain specifics when time for attention is limited

**Table 2.** Carlile’s categories of boundary objects^2,22^.

Category	Description	Example
Syntactic	Shared language as a way of representing knowledge	A mathematical theory
Semantic	Shared meaning that can be transferred	Role, and at times challenge, of interpretation in communication and collaboration
Pragmatic	“Facilitates a process where individuals can jointly transform their knowledge”^22^	Transforming knowledge, co-creating new knowledge, during real-time collaboration

**Table 3.** Examples of boundary and boundary objects across the three waves^16(p143-145)^.

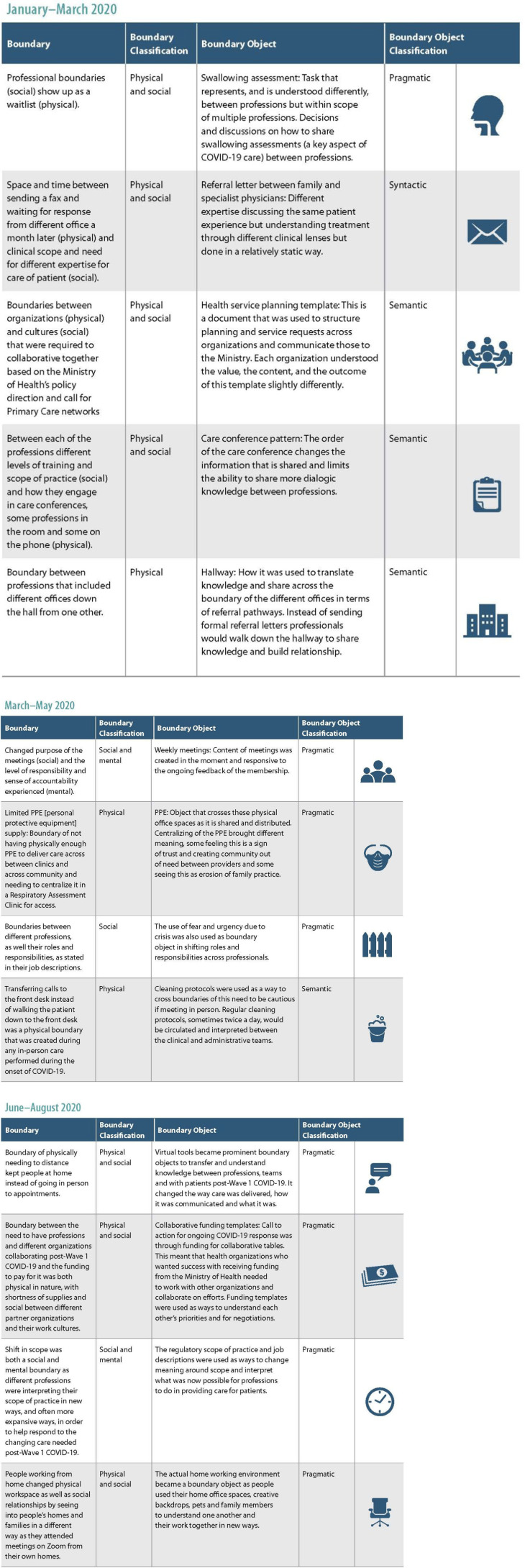

### Boundary objects

Boundary objects are objects used to share knowledge across boundaries.^23^ Boundary objects are used at the site of boundaries to communicate and share knowledge between those on either side of the boundary. They can be physical (e.g., medical records) or conceptual (e.g., patient experience) constructs that provide a focal point for sharing information between two or more sides across a boundary.

The first usage of a boundary object was conceptualized in the 1980s by Star and Griesemer who looked at how scientific work was achieved across scientists and citizens. Because different levels of training bring different ways of thinking and approaching the same topic there was a need for “generalizable findings.”^24^ These objects, through their “interpretive flexibility…afford coordination of activities across diverse social spheres,”^7^ meaning they generate separate and joint action. How objects are used and their instrumental role in professional practice is now better understood; we see that how we organize, know, and learn in the workplace is highly influenced by these objects. Emphasizing local context such as culture, leadership, and also “infrastructural context” ^21^ is important for understanding our collaborations differently. Boundary objects are dynamic, so what is used as a boundary object in one context may not be used in a different context. Identifying boundary objects is a useful tool for seeing the different elements that contribute to the process of collaboration.

A well-known boundary object that has been researched across healthcare sites is personal health records. Not only do these records have different audiences, including consumer and clinical, there are also various professional motivations around using or not using the record.^25^ This use of personal health records supports heterogenous groups with different interests, objectives and patient outcomes to providing care for the patient.

In categorizing the boundary objects during data collection, I applied Carlile’s^22^ three categories to classify the function of boundary objects: syntactic, semantic, and pragmatic.

## Results

One of the interesting aspects of collaboration is that boundaries and boundary objects are continually being shaped, negotiated, and re-created. These three tables show the different boundaries and boundary objects that were traced during the collaborative process of pre-Wave 1 COVID-19; Wave 1 COVID-19; and post-Wave 1 COVID-19.

## Discussion

### Construction of boundaries and boundary objects as observable collaborative process

For the purposes of this article “boundary construction” and “boundary object construction” both refer to the ongoing process that is observable over time as human and non-human actors’ interactions are performed. This is the dynamic process of collaboration in action. Through these interactions, boundaries are created or dissolved and different objects are used to share knowledge across these changing boundaries.^26^ This dynamic or performative coming together in practice is what creates the boundary and boundary objects. The focus of much current research, including this article, is “no longer on boundaries and the frequently difficult task of crossing them, but rather…the continuous process of formation of boundaries between subject and object.”^20^ This article introduces concepts of constructing boundaries and boundary objects used in other disciplines, such as adult education and management, to support our understanding of collaboration in primary care.

### Applying concepts in practice

To apply these theoretical concepts in practice, I invite collaborators to take a pause and consider the boundaries and boundary objects that are actively being constructed in their workplaces. To begin, take a moment and write down a few examples of (1) physical (e.g., walls, roads, rules, guidelines, and scope of practice); (2) social (e.g., ways of distinguishing identity from other groups); and (3) mental (e.g., identify separations in your own mind as to what goes together and what does not such as certain services or care team members) boundaries they experience in their collaborations.^6^ Next, take a moment and write down a list of the people and objects involved in their collaborative experiences. Examples of non-human actors might be doors, personal protective equipment, health records, rules, or scheduled shift changes. After creating a list of objects, consider if there are any listed that have been used as a way to share knowledge across boundaries. You can explore what sort of information or knowledge did you gain from that particular boundary object.

Although counterintuitive, bringing awareness to boundaries can empower team members and partners by improving their approach to collaborative practices and relationships.^16,27^ Across organizations, in healthcare and in other sectors, further understanding the process of collaboration may provide insight into addressing this accountability deficit by adding visibility to multiple micro-decisions and the multiple actors involved. Much research has been done on the need for collaboration, the roles that boundaries and boundary objects can play in supporting collaboration, and the inclusion of non-human actors in collaborations.^18,20,23,28,29^ The contribution of this research is regarding the *process* of collaboration, and this paper brings awareness to the process of creating boundaries and boundary objects for members of healthcare teams so that they can have a new approach to understanding collaboration.

## Conclusion

In collaborative spaces, healthcare professionals, as well as the administrative and executive teams that support them, are continuously working together—coming together, building, crossing, and re-creating boundaries. This is hard work. Often, this part of the process is taken for granted or it is assumed it will just sort itself out. This paper spotlights one way to explore how collaboration on the ground happens by introducing two concepts from other disciplines, the construction of boundaries and boundary objects. Tracing the continual changes of boundaries and boundary objects makes the process of collaboration observable. By understanding a new theoretical and observational way of reframing collaboration, those working in these situations might be better equipped to handle challenges related to collaboration.

The aim of this article was to introduce a process view of collaboration and to prompt new ways to approach collaborative practices in healthcare workplaces. This article positions collaboration as a dynamic process that is constantly being created, re-created, negotiated, and re-negotiated across both human and non-human actors. By better understanding the process of collaboration, this research could potentially improve collaborative outcomes for team members as they strive to enact collaborative policy that is more reflective and efficient. By understanding who and what is collaborating, and the different dynamics that are continuously at play, collaborators are able to adjust how they engage in the process of collaboration, which may change their outcomes. This article argues that if we want an integrated system, one that supports multidisciplinary teams and inter-organizational partnerships, we need to better understand the complex dynamics, structures, partners, and negotiations that go into the planning and implementation of effective collaboration.
